# First person – Callum Rakhit and Ricky Trigg

**DOI:** 10.1242/dmm.038992

**Published:** 2019-02-12

## Abstract

First Person is a series of interviews with the first authors of a selection of papers published in Disease Models & Mechanisms, helping early-career researchers promote themselves alongside their papers. Callum Rakhit and Ricky Trigg are co-first authors on ‘Early detection of pre-malignant lesions in a *KRAS*^G12D^-driven mouse lung cancer model by monitoring circulating free DNA’, published in DMM. Callum is a Clinical Scientist (Bioinformatics) in the lab of Barnaby Clark at Rayne Institute, King's College Hospital, London, UK. His main research interest is identifying predictive factors in large biological datasets using informatic tools. Ricky is a Postdoctoral Research Associate in the lab of Dr Suzanne Turner at the University of Cambridge, Cambridge, UK, investigating mechanisms of resistance to targeted therapies in neuroblastoma.


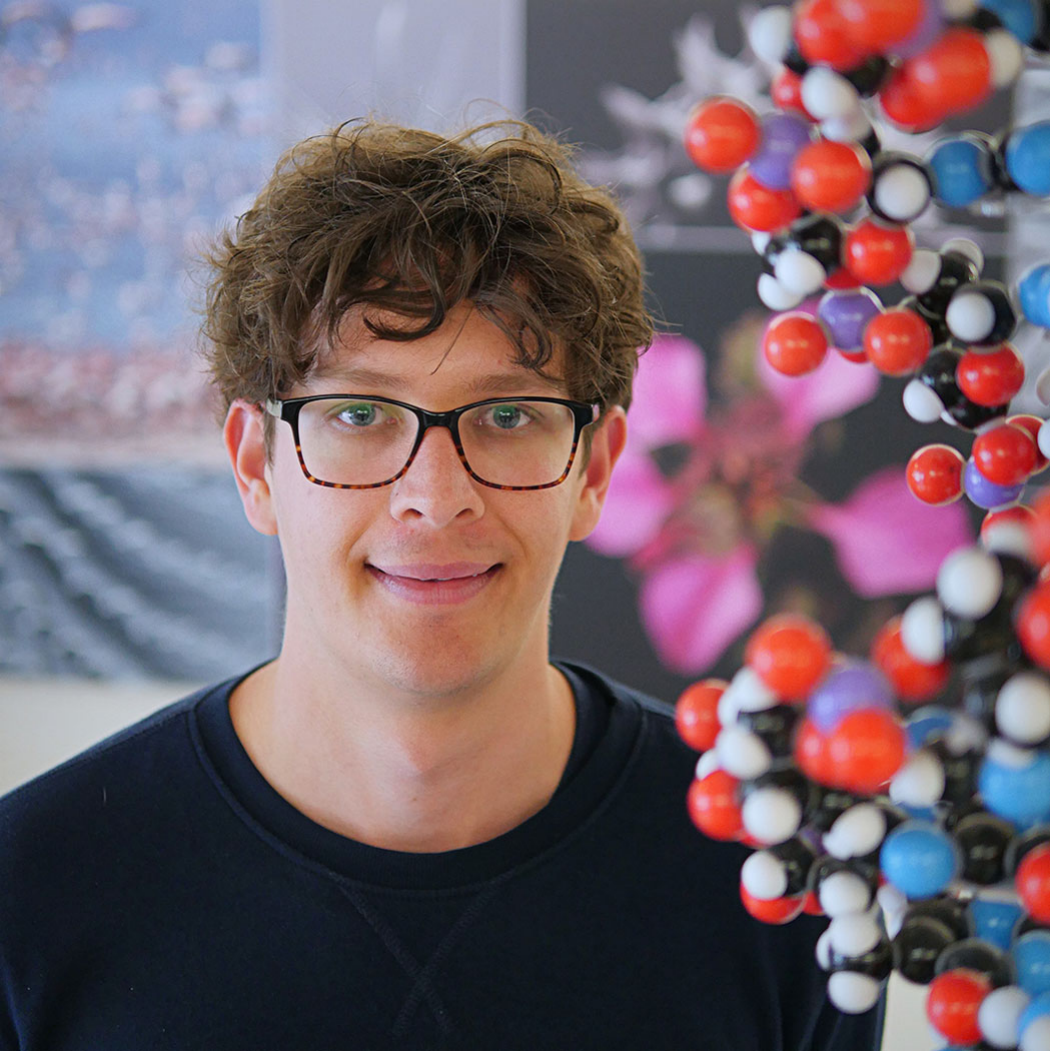


**Callum Rakhit**

**How would you explain the main findings of your paper to non-scientific family and friends?**

Lung cancer is the leading cancer killer worldwide, in part due to its late diagnosis. We modelled pre-cancerous lung tumours in mice to determine whether evidence of these very early-stage tumours could be detected in the blood. Specifically, we were interested in the analysis of DNA in the blood. This DNA, often termed circulating free DNA (cfDNA), is present at low levels in the blood of healthy individuals, but levels are often elevated in patients with cancer, with the extra cfDNA deriving from cells of the patients' tumours. We monitored the development of pre-cancerous tumours in the mice using computed tomography (CT) scans and also took small blood samples to obtain cfDNA. We found that levels of cfDNA rose as the tumours grew in size, as expected. More excitingly, at later time points when the tumours were larger but still pre-cancerous, we detected the mutation that caused the tumours to develop.

**What are the potential implications of these results for your field of research?**

The results from our study suggest that it may be possible to detect pre-cancerous lesions or early-stage cancers in patients by analysing cfDNA. Early detection of cancer based on analysis of cfDNA would be expected to reduce cancer-associated deaths, but would first involve the development and implementation of screening programmes for at-risk individuals. However, further animal studies looking into cfDNA-based detection of pre-cancerous tumours in other tissues must be conducted before the concept can be applied to patients.

“[…] the model faithfully recapitulates some of the pre-cancerous lung tumours that are found in humans, and if the tumours are left to develop over extended time periods, they can become cancerous.”

**What are the main advantages and drawbacks of the model system you have used as it relates to the disease you are investigating?**

Mice are good model organisms in general due to their small size, short gestation time and genetic similarities to humans. We decided to model pre-cancerous lung tumours in mice because these tumours are often asymptomatic and therefore undetected in human patients. By having control over tumour development, we could precisely monitor the growth of the tumours and make comparisons with cfDNA in blood samples at chosen time points. The mouse model that we used has been very well characterised by other scientists, which meant that we could use the model in accordance with published protocols. Most importantly, the model faithfully recapitulates some of the pre-cancerous lung tumours that are found in humans, and if the tumours are left to develop over extended time periods, they can become cancerous. The main drawback of this model is that multiple tumours tend to develop simultaneously, which is not representative of lung tumour development in humans and therefore made the interpretation of our results more complex.

**What has surprised you the most while conducting your research?**

To initiate lung tumour development, we infected mice intranasally with adenoviral Cre recombinase under one of two types of promoter: the cytomegalovirus (CMV) promoter in the case of Ad5-CMV-Cre and a surfactant protein C (SPC) alveolar type II cell-specific promoter in the case of Ad5-mSPC-Cre. Whereas Ad5-CMV-Cre targeted all cells and induced observable bronchial lesions as an artefact of its delivery, Ad5-mSPC-Cre targeted only alveolar type II cells. Therefore, we were not surprised to observe higher tumour burdens over time in Ad5-CMV-Cre-treated mice compared with Ad5-mSPC-Cre-treated mice. However, based on previous reports that intranasal infection of mice AD5-CMV-Cre also targets non-lung cells such as resident myeloid lineage cells, we expected cfDNA levels to be higher in Ad5-CMV-Cre-treated mice compared with Ad5-mSPC-Cre-treated mice. We, in fact, found that mice with similar tumour burdens had similar cfDNA levels independent of the type of adenoviral Cre recombined, suggesting that infection of lung resident myeloid lineage cells and/or the development of bronchial lesions by Ad5-CMV-Cre does not significantly impact the release of cfDNA.


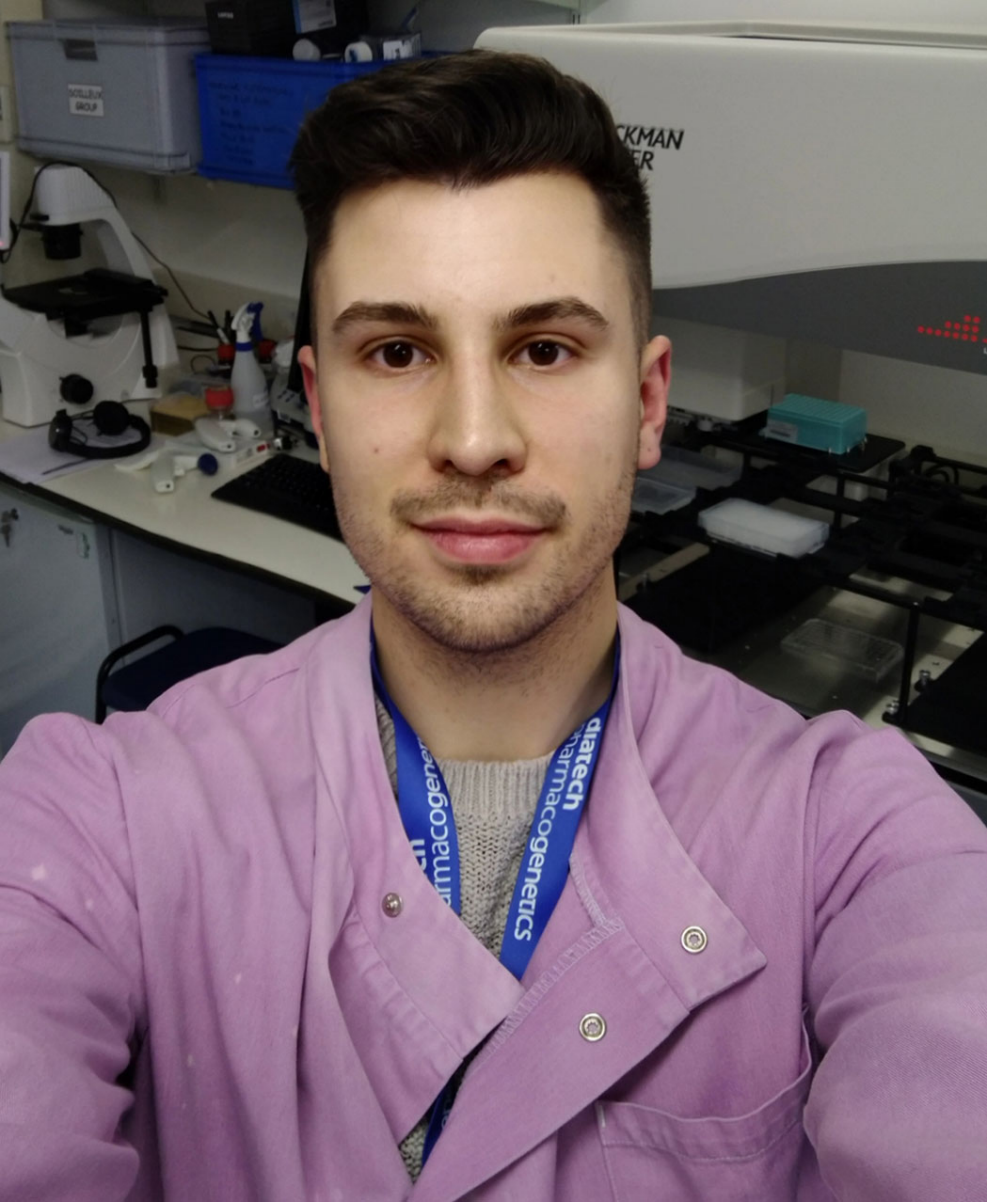


**Ricky Trigg**

“[…] the continued development and adaption of new scientific instrumentation and methodologies, in the areas of extraction, sequence detection and analysis, will be needed to provide ever more reliable and clinically trustworthy cfDNA assays.”

**Describe what you think is the most significant challenge impacting your research at this time and how will this be addressed over the next 10 years?**

cfDNA is highly fragmented, low in abundance, unstable in the circulation and primarily derived from healthy tissue rather than tumours, even in advanced cancer patients. Therefore, the continued development and adaption of new scientific instrumentation and methodologies, in the areas of extraction, sequence detection and analysis, will be needed to provide ever more reliable and clinically trustworthy cfDNA assays. This may be achieved through improved droplet digital PCR methodologies, improved isolation and enrichment protocols for tumour-derived cfDNA, or improved accuracy levels and reduced DNA input requirements from next-generation sequencing technologies.
